# Rethinking Manure Application: Increase in Multidrug-Resistant *Enterococcus* spp. in Agricultural Soil Following Chicken Litter Application

**DOI:** 10.3390/microorganisms9050885

**Published:** 2021-04-21

**Authors:** Dorcas Oladayo Fatoba, Akebe Luther King Abia, Daniel G. Amoako, Sabiha Y. Essack

**Affiliations:** 1 Antimicrobial Research Unit, College of Health Science, University of KwaZulu-Natal, Private Bag X54001, Durban 4000, South Africa; amoakodg@gmail.com (D.G.A.); essacks@ukzn.ac.za (S.Y.E.); 2 Department of Medical Microbiology, School of Laboratory Medicine and Medical Sciences, University of KwaZulu-Natal, Durban 4000, South Africa

**Keywords:** animal manure, antibiotic resistance, *Enterococcus* spp., chicken litter, environmental reservoirs, multidrug resistance, public health, agricultural soil

## Abstract

The current study investigated the impact of chicken litter application on the abundance of multidrug-resistant *Enterococcus* spp. in agricultural soil. Soil samples were collected from five different strategic places on a sugarcane farm before and after manure application for four months. Chicken litter samples were also collected. Enterococci were enumerated using the Enterolert^®^/Quanti-Tray 2000^®^ system and confirm and differentiated into species using real-time PCR. The antibiotic susceptibility profile of the isolates was determined using the disk diffusion method following the European Committee on Antimicrobial Susceptibility Testing (EUCAST) guidelines. The overall mean bacterial count was significantly higher (*p* < 0.05) in manure-amended soil (3.87 × 10^7^ MPN/g) than unamended soil (2.89 × 10^7^ MPN/g). Eight hundred and thirty-five enterococci (680 from soil and 155 from litter) were isolated, with *E. casseliflavus* being the most prevalent species (469; 56.2%) and *E. gallinarum* being the least (16; 1.2%). Approximately 56% of all the isolates were resistant to at least one antibiotic tested, with the highest resistance observed against tetracycline (33%) and the lowest against chloramphenicol (0.1%); 17% of *E. faecium* were resistant to quinupristin-dalfopristin. Additionally, 27.9% (130/466) of the isolates were multidrug-resistant, with litter-amended soil harbouring more multidrug-resistant (MDR) isolates (67.7%; 88/130) than unamended soil (10.0%; 13/130). All isolates were susceptible to tigecycline, linezolid and gentamicin. About 7% of the isolates had a multiple antimicrobial resistance index > 0.2, indicative of high antibiotic exposure. Although organic fertilizers are regarded as eco-friendly compared to chemical fertilizers for improving soil fertility, the application of untreated animal manure could promote the accumulation of antibiotics and their residues and antibiotic-resistant bacteria in the soil, creating an environmental reservoir of antimicrobial resistance, with potential human and environmental health risks.

## 1. Introduction

Poor soil fertility is a significant challenge for small and large-scale farming systems in sub-Saharan Africa, and chemical and organic fertilizers are frequently added to soil to improve its quality, texture, and crop yield [1]. However, chemical fertilizers affect beneficial microorganisms in the soil, cause an imbalance in soil pH, contaminate groundwater through leaching without fully benefiting plants, and cause plant disease [2]. On the other hand, organic manure adds nutrient-rich organic matter, which improves soil fertility, texture, water-holding capacity, and imparts resistance to wind and water erosion [3,4]. Thus, applying animal manure to soil has become common in agricultural farms in many countries, including South Africa [1,5], as it improves soil properties and increases productivity [6].

Although the use of antibiotics in livestock farming has proven to be beneficial for economic reasons, their use as growth promoters for prophylaxis, metaphylaxis and treatment establishes a reservoir of antibiotic-resistant bacteria (ARB), including multidrug-resistant (MDR) ones and antibiotic resistance genes (ARGs) in the gastrointestinal tract of livestock, and subsequently their waste [7]. The addition of such animal waste as manure to the soil, without treatment, may contribute to the transmission of antibiotic resistance to soil bacteria and pose serious environmental risks [8]. This agricultural practice has resulted in the contamination of soil, surface water, groundwater, and the food chain with antibiotic residues and ARB, posing a severe public health concern associated with farm produce such as raw vegetables [5,9].

*Enterococci* are Gram-positive natural commensals inhabiting humans and animals’ digestive systems with a wide range of species such as *E. faecalis*, *E. faecium*, *E. casseliflavus*, *E. gallinarum*, *E. durans*, *E. munditi*, *E. hirae*, and *E. avium* [10]. The abundance of *Enterococcus* spp. in animal and human faeces and their prolonged survival in the environment have made them a popular indicator of faecal contamination in the environment [11,12]. Although considered a commensal in humans, certain *Enterococcus* species have been identified as high-ranking (second to staphylococci) agents causing nosocomial infections in humans [13]. These bacteria, especially multidrug resistance ones, in animal manure applied to agricultural fields represent a significant environmental and public health concern that needs considerable attention through continuous monitoring. 

Therefore, this study investigated the prevalence of antibiotic-resistant *Enterococcus* spp. in soil amended with chicken litter on a sugarcane farm in KwaZulu-Natal, South Africa. Such information would guide decision making regarding the use of manure and emphasise the importance of antibiotic stewardship in agricultural practices, thus protecting human and environmental health.

## 2. Materials and Methods

### 2.1. Ethical Clearance

This study was part of a larger project for which ethical approval had been received from the Animal Research Ethics Committee (Reference: AREC073/016PD) and the Biomedical Research Ethics Committee (Reference: BCA444/16) of the University of KwaZulu-Natal. The study was also approved by the South African National Department of Agriculture, Forestry, and Fisheries (Reference: 12/11/1/5 (879).

### 2.2. Study Location

**Figure 1:**
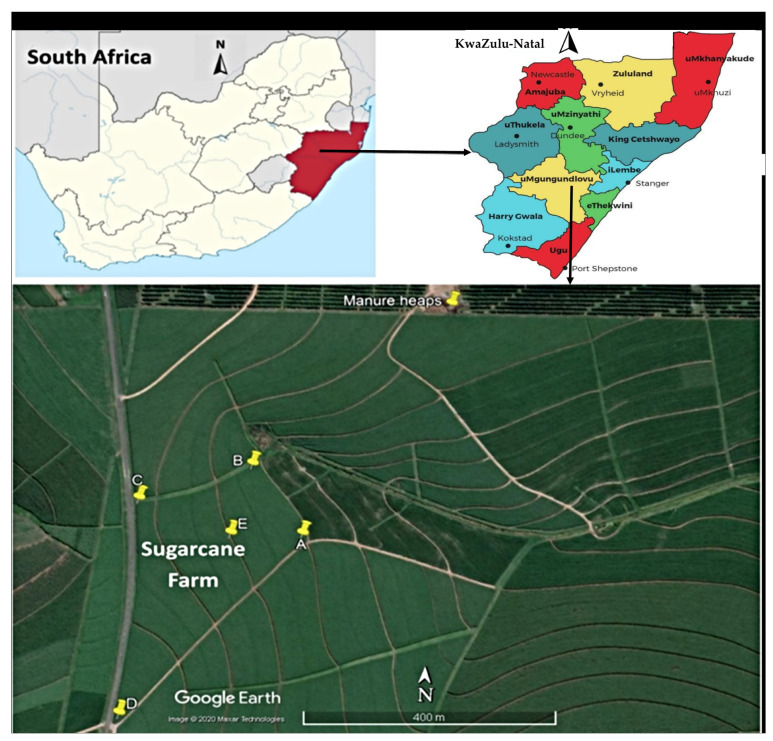
Map of the study site, including the sampling points (A, B, C, D, E and manure heap). Source: Google Earth.

This study was carried out for four months, between October 2018 and February 2019, on a sugarcane farm in the uMgungundlovu District, KwaZulu-Natal, South Africa (Figure 1). The district has a population of 1,069,657, with a population density of 110.7 persons/km^2^. The area is home to the bulk of food animal production firms (pigs and poultry) and agriculture, mainly sugarcane, pear, and vegetable farms. Some of the water bodies around the farm in this locality include Mqeku, Mkabela, Mbhava, and Sterkspruit, which are small rivers that drain into the main Umgeni River (the primary source of drinking water for the people living in Pietermaritzburg). The sugarcane is planted between September and November and becomes fully mature in about 12 to 14 months. Soil amendment with chicken litter (a mix of chicken manure and wood shavings) is a common agricultural practice in the locality because of its availability, cost-effectiveness, and efficiency in improving soil quality. Chicken litter is usually randomly spread over the soil surface about ten days before planting. Urea is also applied to the soil seven days after manure application.

### 2.3. Sample Collection

Soil samples were collected from five points of the sugarcane farm (Figure 1) on days 1, 2, 3, 5, and 9 before manure application. Following manure application, samples were collected on the day of application (day 0), then days 1, 3, 7, 14, 21, and bi-monthly after that for three months after the chicken litter application. Samples were collected until the farm became inaccessible due to the height of the plants. 

Using a sterile hand shovel, ≈50 g of soil was aseptically collected within the top 5 cm of the soil and transferred into sterile ziplock bags. The same quantity of chicken litter was also collected from a heap of unapplied manure around the farm (Figure 1). All the soil samples were collected in duplicates and the chicken manure in triplicates. Samples were transported on ice packs to the laboratory for analyses within 6 h from collection. A total of 275 samples (82 chicken litter and 193 soil) were collected throughout the study.

### 2.4. Sample Processing and Enumeration of Enterococcus spp.

The Enterolert^®^-18^®^ Quanti-Tray^®^/2000 system (IDEXX Laboratories (Pty) Limited, Totowa, NJ, United States) was used to enumerate *Enterococcus* spp. Soil and chicken litter samples were processed for analysis, as previously described by Abia et al. [14]. Briefly, 5 g of sample was transferred from a well-shaken zip-lock bag into a sterile bottle containing 5 mL of sterile distilled water, giving a 1:1 (*v*/*v*) dilution. The mixture was shaken vigorously to dislodge the bacteria from the soil into the water. The bottle was allowed to settle for 20 min, and 1 mL of the supernatant was extracted, topped up to 100 mL with sterile distilled water, and processed following the IDEXX protocol for water sample analysis (IDEXX Laboratories (Pty) Ltd., Johannesburg, South Africa). Ten positive wells in the quanti-tray (those that fluoresced under the UV light) were randomly picked, and their content was streaked onto Bile Aesculin agar plates (Lab M, Lancashire, UK) and incubated at 44 ± 0.5 °C for 24 h to obtained pure colonies. One pure isolate was collected per plate and stored in Trypticase soy broth (Oxiod, Hampshire, England) with 20% glycerol at −80 °C for further analysis.

### 2.5. DNA Extraction, Molecular Confirmation and Differentiation Enterococcus Species

Stored isolates were resuscitated by culturing them on nutrient agar (Lab M, Lancashire, UK) at 41 °C for 24 h. Colonies were then transferred to sterile Eppendorf tubes containing 200 µL of sterile distilled water and the DNA was extracted using the boiling method as previously described [15]. The supernatant was then used as the DNA template for the PCR assays. 

Real-time polymerase chain reaction (RT-PCR) was used to confirm the isolates to genus level and distinguish between the species on a QuantStudio^®^ 5 Applied Biosystems (Applied Biosystems, ThermoFisher, Waltman, MA, USA) real-time PCR machine. The confirmation to genus level was carried out by targeting the *tuf* gene [16], using cycling conditions previously described by Molechan et al. [17]. All the confirmed *Enterococcus* isolates were further screened to speciate them as *E. casseliflavus*, *E. faecalis*, *E. faecium*, and *E. gallinarum*, using species-specific primers (Table S1) and PCR conditions previously described [17]. *Enterococcus* isolates that did not fall within the four species categories were tagged as *Enterococcus* spp. All primers were supplied by Inqaba Biotech Industries Ltd., Pretoria, South Africa. All reactions contained a positive control (Table S1) and a No Template Control (reaction mixture but no DNA). Melt curves were analysed using the QuantStudio^TM^ Design and Analysis Software v.1.3.1 (Applied Biosystems, ThermoFisher Waltman, MA, USA).

### 2.6. Antibiotic Susceptibility Testing

The confirmed *Enterococcus* isolates were subjected to antibiotic susceptibility testing using the disk diffusion method on Mueller–Hinton agar (Lab M, Lancashire, UK) according to the Clinical and Laboratory Standards Institute [18] and the European Committee on Antimicrobial Susceptibility Testing (EUCAST) [19] for breakpoints absent in the CLSI guidelines. The isolates were tested against 16 antibiotics in 12 antibiotic classes. These included ampicillin (AMP, 10 µg), teicoplanin (TEC, 30 µg), vancomycin (VAN, 30 µg), streptomycin (STR, 300 µg), linezolid (LZD, 30 µg), imipenem (IPM, 10 µg), erythromycin (ERY, 15 µg), ciprofloxacin (CIP, 5 µg), levofloxacin (LEV, 5 µg), nitrofurantoin (NIT, 300 µg), gentamicin (GEN, 120 µg), chloramphenicol (CHL, 30 µg), sulfamethoxazole-trimethoprim (SXT, 25 µg), tetracycline (TET, 30 µg) and tigecycline (TGC, 15 µg) (Oxoid, Hampshire, England). *E. faecium* was additionally tested against quinupristin-dalfopristin (QD, 15 µg). *E. faecalis* ATCC 29,212 was used as a positive control. The inhibition zones’ diameters were measured in millimetres and interpreted as susceptible (S), intermediate (I), or resistant (R) [18]. EUCAST was used for three antibiotics (TGC, 15 µg, SXT, 25 µg, and IMP, 10 µg). Isolates resistant to one or more antibiotics in three or more different antibiotics classes were classified as MDR.

The multiple antibiotic resistance index (MARI) of each isolate was calculated as a/b (a: number of antibiotics to which the isolates were resistant, b: number of antibiotics against which the isolates were tested) [20]. The MARI of each sample group was calculated using the formula a/(bc), where “a” represents the aggregate antibiotic resistance score of all *Enterococcus* isolates from each sample group, “b” is the number of antibiotics tested against the isolates, and “c” represents the total number of isolates per sample group [20].

### 2.7. Data Analysis

All statistical analyses were performed using Microsoft Excel 2016 and the Statistical Package for the Social Science (SPSS v26, IBM Corporation, Armonk, NY, USA). Before analysis, enterococci counts were log-transformed, and the geometric means were used to describe the microbial concentration in soil and chicken litter. To calculate the log counts and the geometric means, all values > 2419.6 were approximated to the nearest whole number (2420), and values < 1 were considered as 1. One-way analysis of variance (ANOVA) and the Games–Howell post hoc test were conducted to compare the mean *Enterococcus* counts and the number of antibiotic-resistant species between the chicken litter, litter-amended soil and soil samples collected before litter application. Results were considered statistically significant if the *p*-value was <0.05.

## 3. Results

### 3.1. Quantification of Enterococcus spp.

**Figure 2:**
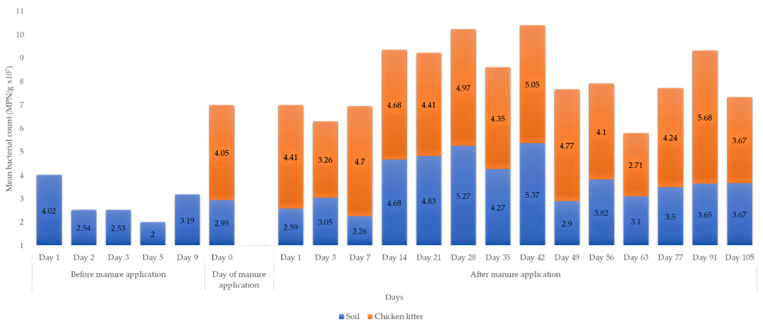
Mean *Enterococcus* spp. count per sampling source and sampling day. No litter sample collected before the litter application. The farm was burnt on day 2 in preparation for harvest. Rainfall events were recorded on day 3 (before litter application) and day 14, 42 and 77 after the litter amendment. Urea was applied to the farm on day 7.

All the soil and chicken litter samples from the various sample groups and points tested positive for *Enterococcus*. The highest mean count (5.68 × 10^7^ MPN/g) per sample round was observed in the chicken litter (Table S2). The overall mean bacterial count was higher in litter-amended soil (3.87 × 10^7^ MPN/g) than unamended soil (2.89 × 10^7^ MPN/g) (Figure 2).

There was an overall statistically significant difference (*p* = 0.000; *p* < 0.05) in *Enterococcus* count between the three sample groups (Table 1). Games–Howell post hoc test indicated that the overall *Enterococcus* mean count in the chicken litter was significantly higher than the litter-amended soil (*p* = 0.01, *p* < 0.05), and unamended soil (*p* < 0.001, *p* < 0.05). A statistically significantly higher mean *Enterococcus* count was observed in the litter-amended soil than unamended soil (*p* = 0.01, *p* < 0.05).

### 3.2. Prevalence of Enterococcus spp. Isolates in Soil and Chicken Litter

**Figure 3:**
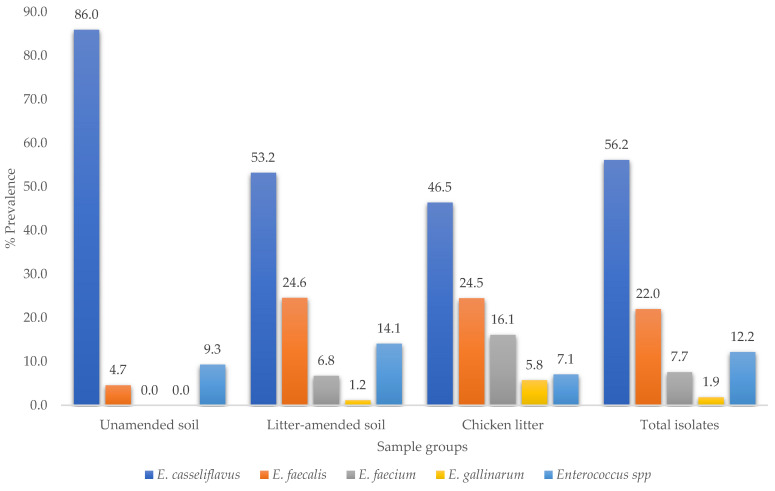
Distribution of *Enterococcus* spp. across sample types.

A total of 835 enterococci (680 from soil and 155 from chicken litter) isolates were confirmed. *E. casseliflavus* was the most prevalent species (56.17%), and *E. gallinarum* as the least prevalent (1.9%) (Figure 3). *E. faecium* and *E. gallinarium* were not detected in unamended soil samples; 12.2% of the isolates could not be classified into any of the four *Enterococcus* species.

### 3.3. Antibiotic Susceptibility Profiles of Enterococcus spp.

**Figure 4:**
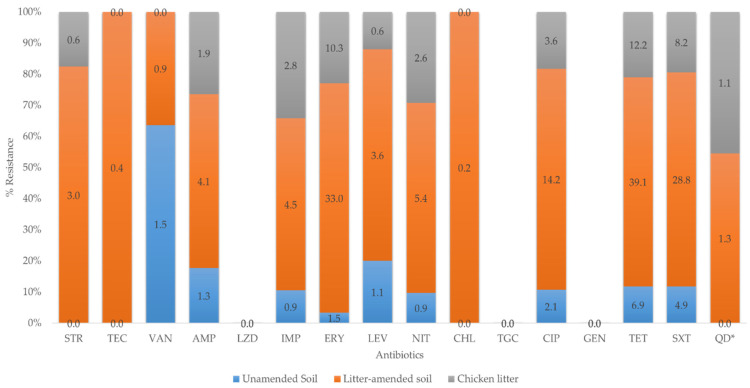
Prevalence of antibiotic-resistant *Enterococcus* in the soil and chicken litter. AMP = ampicillin, TGC = tigecycline, TET = tetracycline, CHL = chloramphenicol, GEN = gentamicin, TEC = teicoplanin, VAN = vancomycin, STR = streptomycin, LZD = linezolid, IPM = imipenem ERY = erythromycin, CIP = ciprofloxacin, LEV = levofloxacin, NIT = nitrofurantoin, SXT = sulfamethoxazole/trimethoprim, QD = quinupristin-dalfopristin. QD* is reported only for *E. faecium* isolates.

Overall, 466 (55.8%) of 835 *Enterococcus* isolates in this study were resistant to at least one antibiotic, of which 321 (68.9%) were from litter-amended soil, 93 (19.9%) from chicken litter, and 52 (11.2%) from unamended. Overall, the highest resistance observed was against tetracycline (58.2%) and the lowest against chloramphenicol (0.2%) (Figure 4). None of the isolates were resistant to tigecycline, linezolid, and gentamicin. The variation in the number of resistant isolates with sampling days is shown in Figure S1 (Supplementary Materials). The susceptibility profile of quinupristin-dalfopristin was only reported for *E. faecium*, and 19% were resistant isolates. Only *E. casseliflavus* (2%) and *E. gallinarum* (4%) species showed resistance to vancomycin while *E. faecalis* (7%) were of intermediate susceptibility (Table S3; Supplementary Materials).

Although there was an overall increase in the number of resistant isolates following the chicken litter amendment, this was not statistically significant (Table 2) in the number of resistant isolated between the three sample groups. 

### 3.4. Prevalence of Multidrug Resistance and Calculation of MARI

Multidrug resistance was observed in 27.8% (130/466) of the resistant enterococci isolates. Among these MDR, the litter-amended soil isolates had the highest percentage, 67.7% (88/130), followed by the chicken litter 22.3% (29/130) and unamended soil 10% (13/130) (Table S3). The division of the MDR into species revealed that *E. faecium* had the highest rate (41%, 26/64) of MDR compared to other species, with the least MDR observed in *E. faecalis* (13%, 23/184).

Overall, 63 MDR patterns were observed across the enterococci isolates, the most prevalent phenotype being ERY-TET-SXT (Table S3). At the species level, *E. casseliflavus*, *E. faecium*, *E. faecalis*, other *Enterococcus* spp. and *E. gallinarum* showed 40, 23, 12, and 12 MDR patterns, respectively. 

**Figure 5:**
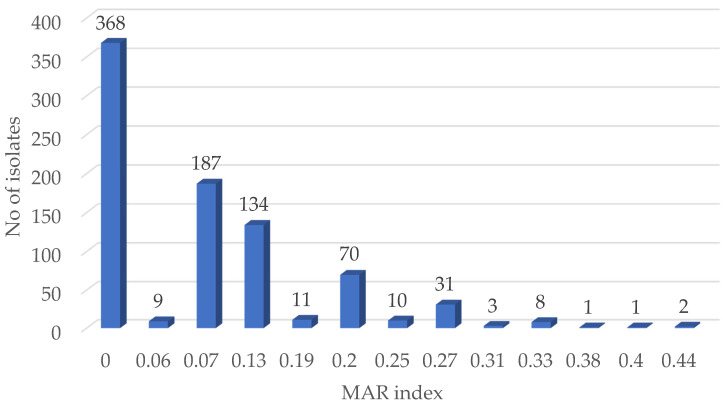
Multiple antibiotic resistance (MAR) indices of all the *Enterococcus* isolates.

The isolates’ MAR indices ranged from 0.13 (resistance to two antibiotics) to 0.44 (resistance to seven antibiotics) (Figure 5). In total, 12.1% (56/466) of the resistant isolates had a MARI > 0.2. Of these, 58.9% (33/56) was from the litter-amended, 26.8% (15/56) of the isolates from the chicken litter, and 14.3% (8/56) from the soil before the litter amendment. The average MAR indices value according to the sample groups revealed that the chicken litter had the highest value of 0.09 compared to the litter-amended soil (0.08) and the soil samples before litter-amendment (0.06) (Table S4).

## 4. Discussion

Although animal manure is regarded as an organic approach to soil fertility improvement, its application to soil may introduce numerous ARB to the environment. Thus, we investigated the impact of chicken litter application on the prevalence of antibiotic-resistant *Enterococcus* spp. in agricultural soils. There was a marked increase in *Enterococcus* counts in the soil following manure application, with some species only identified in manure-amended soils and chicken litter but not in the soil before manure application. The *Enterococcus* spp. were resistant to tetracycline, erythromycin, trimethoprim-sulfamethoxazole, and fluoroquinolone, commonly used in poultry production in South Africa. Approximately 28% of the resistant enterococci were MDR, with a substantial percentage of them having a MARI > 0.2.

### 4.1. Enumeration of Enterococcus before and after Manure Application

*Enterococcus* has been widely used as a faecal bacterial indicator in the environment. In the current study, the mean *Enterococcus* count in the litter-amended soil was statistically significantly higher than in the soil before amendment (Table 1), indicating that manure application impacted the soil bacterial abundance. This could have happened in two ways. Firstly, chicken manure is exceptionally nutrient-rich, and its application on the farm resulted in the enrichment of indigenous bacteria, including *Enterococcus*. The use of animal manure for soil fertilization has been shown to enhance resident soil bacteria’s proliferation in agricultural soil [21]. Secondly, *Enterococcus* is a normal flora of human and animal intestines [11,12]. Therefore, manure application resulted in the direct introduction of *Enterococcus* into the soil. The second argument is supported by the fact that the chicken litter recorded a statistically significantly higher bacterial count than the soil before manure application and manure-amended soil. Consistent with our findings, Marti and colleagues [21] reported that soil fertilisation with swine and dairy manure increased the count of viable bacteria in the soil.

Although there were fluctuations in the *Enterococcus* counts during the different sampling rounds after chicken litter application, an overall decline to baseline values was observed by the last sampling date (day 105). Some studies have indicated that bacteria of animal manure origin only survive in the soil environment for a short period (days to few months) [22,23,24], while others have indicated that enterococci could persist in manured soil environments for up to a year [25]. Cools et al. [24] demonstrated that *Enterococcus* spp. derived from pig manure could survive in the soil for 54 days in a study conducted in Belgium. Contrarily, [25] reported in a USA study that enterococci concentration in the swine manured soil decreased to concentrations equivalent to the no-manure soil after one year of manure amendment. Therefore, although the sampling in the current study ended on day 105, the manure’s effect could be felt far beyond the sampling period. This could explain the presence of *Enterococcus* in the soil before manure application in this study. The long-term persistence of enterococci in the litter-amended soil is worrying, as the potentially pathogenic strains could enter the food chain or get washed during rainfall events to nearby surface water bodies. This also implies that yearly manure application could maintain high enterococci concentrations in the soil environment, with potential environmental and human health implications.

### 4.2. Prevalence of Different Enterococcus Species

According to species, the molecular characterization of the enterococci revealed differences between the soil before and after the litter amendment. Although *E. casseliflavus* was present in all sample sources, its high prevalence and dominance in the soil before manure was expected as this species is plant-associated [26]. Similar findings had earlier been reported in the USA [27]. Contrarily, there was a low *E. faecalis* prevalence in the soil before manure (Figure 3). No other species were identified in this soil. However, after manure application, the prevalence increased in the soil, indicating the manure’s impact. Most importantly, *E. faecium* and *E. gallinarum* were only identified in the litter-amended soil. These species were absent in soil before manure application but present in the chicken-litter samples, further highlighting the chicken litter’s significant impact on the soil *Enterococcus* abundance. These observations corroborate a field experiment in China, which observed a significant increase in bacterial diversity in soil following chicken manure amendment [28]. The appearance of *E. faecium* and *E. gallinarum* as well as the increased detection of *E. faecalis*, which are characteristic of warm-blooded animals in the soil after the litter amendment, implies that these two important human infectious agents may be potential indicators of transmission dynamics between the soil environment and humans, directly through exposure to contaminated soil or indirectly through consumption of poorly washed crops from manure fertilized farms.

### 4.3. Antibiotic Resistance of Enterococcus Species

Antibiotic-resistant *Enterococcus* spp. was detected in all the sample groups with the highest resistance percentage in the litter-amended soil (Figure 3). The isolates from the soil before amendment were mostly resistant to tetracycline and trimethoprim-sulfamethoxazole, while the litter-amended soil and chicken litter isolates expressed high-level resistance to tetracycline, erythromycin, and trimethoprim-sulfamethoxazole. The extensive administration of sub-therapeutic doses of antibiotics overtime in food animal production creates a selective advantage for the emergence of ARB in their intestines, which invariably ends up in manure and the environment [29,30,31]. This was reflected in the high percentage resistance to tetracycline, erythromycin, and trimethoprim-sulfamethoxazole among the litter-amended soil and the chicken litter enterococci isolates in the current study. These antibiotics or their homologues are used in the South African poultry industry [32], despite the prohibited use of several critically important antibiotics for humans in other parts of the world such as Europe [33]. The increased frequency of erythromycin-resistant enterococci in the soil after the litter amendment and the high intermediate susceptibility of the isolates to erythromycin and ciprofloxacin observed could have resulted from the addition of tylosin and enrofloxacin to the chicken’s feed and water, and their use for treating infections in the animals [17,32]. A previous study on antibiotic-resistant enterococci in chicken litter in Canada reported a higher prevalence of resistance to tetracycline and ciprofloxacin [34], attributed to the large quantities of antibiotics used for growth promotion in broiler chicken farms [34]. Similarly, a previous study of antibiotic-resistant *Enterococcus* spp. from farm-to-fork conducted in uMgungudlovu District, South Africa, also indicated a higher level of resistance to tetracycline and erythromycin and high intermediate resistance to ciprofloxacin [17]. 

The literature has shown that animals excrete as much as 90% of the antibiotics administered orally or added to the feeds through faeces or urine [35]. Although the use of chicken litter as organic fertilizer is a common agricultural practice in South Africa [1], no previous study has examined the impact of chicken litter application on soil antimicrobial-resistant microorganisms in South Africa. In the current study however, the litter amendment increased the number of antibiotic-resistant enterococci in the soil, which could be attributed to the enrichment of resident resistant enterococci in the soil and the addition of resistant species directly to the soil. This is supported by the increase in the number of resistant enterococci in the soil on the day of chicken litter application (Figure S1; Supplementary Materials). Previous studies have reported animal manure as a reservoir of ARB and a source of environmental (soil) contamination with ARB [9,26]. For example, a study on agricultural soil fertilized with swine manure in Iowa State, USA, showed that the concentration of antibiotic-resistant enterococci in the soil with manure was greater than the control soil that was not treated with manure [25]. Similarly, a practical survey of ARB in chicken manure-amended soil and manure-free soil carried out in China reported a significantly higher prevalence of cultivable ARB in the manure-amended soil than the count in the manure-free soil samples [8].

Although the prevalence of resistant *Enterococcus* spp. was generally higher in the litter amended soil, this was not statistically significant (Table 2), as some external factors played a significant role. For example, a major reduction in the number of resistant *Enterococci* was observed on day 7 (after the chicken litter amendment), which happened to be the day urea was applied to the field. Urea application has been shown to decrease the soil microbial population and diversity [36,37]. The number of antibiotic-resistant enterococci in the litter-amended soil decreased to levels comparable to the soil resistance before amendment at 28 days after the chicken litter amendment, suggesting the depletion of manure nutrient that enhanced the growth of resident bacteria and the death of the litter-borne enterococci [38].

It should, however, be noted that there was also a high tetracycline and trimethoprim-sulfamethoxazole percentage resistance observed in the soil before the chicken litter amendment. These antibiotics or their residues should also be considered regarding the selection pressure they may exert on soil bacterial populations. While some of the antibiotics such as erythromycin and tylosin completely biodegrade in soil within 30 days at 20 °C to 30 °C, only a small amount of antibiotics such as ciprofloxacin, tetracycline, and some sulphonamides degrade even after 30 to 80 days [35,39,40].

The MARI is used in differentiating between bacteria from low- and high-health risk sources. A MARI value greater than 0.2 indicates that such bacterial isolates originate from a source with high antibiotics use contamination [20]. The impact of manure on the presence of antibiotic-resistant *Enterococcus* spp. was further demonstrated here as isolates from the litter-amended soil had a higher percentage of isolates with a MARI > 0.20. Additionally, although the sample groups’ MAR indices were <0.20, the comparable MAR index of the chicken litter (0.09) and the litter-amended soil (0.08) suggests the transmission of ARB from sources of frequent antibiotic use, such as on intensively produced chicken as was the litter source here. Furtula and colleagues [34] reported high average MAR indices for the enterococci isolated from the chicken litter samples and attributed their observation to different antibiotic usage levels in the poultry systems from which the litter was sourced.

## 5. Conclusions

Chicken litter application increased the abundance and diversity of *Enterococcus* species in agricultural soil. There was also an increase in antibiotic-resistant enterococci species, including MDR ones, in the litter-amended soil, suggesting the possibility of the transfer of ARB in the chicken litter to the soil. This was further supported by the overall higher MARI of litter-amended soil than unamended soil. A higher percentage resistance was observed against tetracycline, erythromycin, and sulfamethoxazole-trimethoprim. A substantial number of the isolates from chicken litter shared similar resistance patterns to litter-amended soil isolates, suggesting a possible transfer of ARB (or ARGs) of chicken litter to the agricultural soil. The persistence of antibiotic-resistant enterococci species in the manured soil and the heap of chicken litter throughout this study highlights the risk of antibiotic resistance exposure when humans and animals consume contaminated farm produce. This study delineated chicken litter as a “hotspot” of antibiotic-resistant enterococci species that can contaminate the soil fertilized with it and pose a public health threat from its incorporation into plants, run-off to water sources and direct contact in occupationally exposed workers. It is, therefore, necessary to rethink the use of animal manure for soil fertilization. Since composting has been reported to reduce the number of ARB in animal manure, this should be carried out on chicken litter before its application to the soil to minimize soil contamination with ARB and reduce the possible dissemination of antibiotic resistance from chicken to farm produce. Policies on the prudent use of antibiotics in animal production is also required.

## Figures and Tables

**Table 1:** Statistical comparison of mean *Enterococcus* counts between sample sources.

Group	N	Overall Mean *Enterococcus* Count (MPN/g × 10^7^)	Overall *p*-Value	Pair Wise Comparison (Games–Howell)		95% Confidence Interval for Mean (log MPN/g)		*p*-Value
					Mean Difference (±SE)	Lower Bound	Upper Bound	
SBL	48	2.89 (±0.92) *	0.000 ***	SBL vs. LAS	−0.98 (±1.18) *	−1.40	−0.57	0.000 *
LAS	145	3.87 (±1.43) *		SBL vs. CL	−1.52 (±0.19) *	−1.98	−1.06	0.000 *
CL	44	4.41 (±0.92) *		LAS vs. CL	−0.54 (±1.18) *	−0.97	−0.10	0.011 *

* *p* < 0.05; *** *p* < 0.001; SBL: soil before litter application; LAS: litter-amended soil; CL: chicken litter.

**Table 2:** Statistical comparison of the prevalence of resistant-*Enterococcus* isolates between sample sources.

Group	N	Overall Mean Difference (±Standard Deviation) of Resistant *Enterococcus*	Overall ANOVA *p*-Value	Pairwise Comparison (Games–Howell)		95% Confidence Interval for Mean		*p*-Value
						Lower Bound	Upper Bound	
					Mean Difference (±Standard Error)			
SBL	107	0.49 (±0.50)	0.184	SBL vs. LAS	−0.08 (±0.53)	−0.20	0.05	0.324
LAS	573	0.56 (±0.50)		SBL vs. CL	−0.11 (±0.63)	−0.26	0.03	0.165
CL	155	0.60 (±0.49)		LAS vs. SBL	0.08 (±0.53)	−0.05	0.20	0.324

## Data Availability

All data have been included in this manuscript.

## Supplementary Materials

The following are available online at https://www.mdpi.com/2076-2607/9/5/885/s1, Figure S1: Variation in antibiotic-resistant *Enterococcus* to at least one antibiotic throughout sample collection, Table S1: List of genus and species-specific primers and control strains used in this study, Table S2: Enumeration of *Enterococcus* in soil and chicken litter over the sampling period, Table S3: Multidrug-resistant pattern of the *Enterococcus* spp. isolates, Table S4: Percentage of *Enterococcus* isolates that were resistant to at least one antibiotic at each sample point and the multiple antibiotic resistance index.
