# Supplementary material for: Rethinking Manure Application: Increase in Multidrug-Resistant Enterococcus spp. in Agricultural Soil Following Chicken Litter Application
Source: Microorganisms. 2021 Apr 21;9(5):885. doi: 10.3390/microorganisms9050885 (PMC8170873; doi:10.3390/microorganisms9050885)
Supplement: Supplementary file 1 [file microorganisms-09-00885-s001.zip › microorganisms-1167736-SI.pdf]

microorganisms

MDPI

Supplementary materials

Rethinking manure application: increase in multidrug-resistant *Enterococcus* spp. in agricultural soil following chicken litter application

Dorcas Oladayo Fatoba [1,2]*, Akebe Luther King Abia [1], Daniel G Amoako [1,] and Sabiha Y. Essack [1]

[1] Antimicrobial Research Unit, College of Health Science, University of KwaZulu-Natal, Private Bag X54001, Durban, 4000, South Africa; dorcas4c@gmail.com (D.O.F.); lutherkinga@yahoo.fr (A.L.K.A.); amoakodg@gmail.com (D.G.A.); essacks@ukzn.ac.za (S.Y.E.)
[2] Department of Medical Microbiology, School of Laboratory Medicine and Medical Sciences, University of KwaZulu-Natal, South Africa

* Correspondence: dorcas4c@gmail.com

Table S1. List of genus and species-specific primers and control strains used in this study

Control Strain	Primer	Primer sequence 5′-3′	Product size (bp)	Reference
E. faecalis ATCC 51299	ENT1	TACTGACAAACCATTCATGATG	112	(Ke et al., 1999)
	ENT2	AACTTCGTCACCAACGCGAAC		
E. faecalis ATCC 51299	FA1	ACTTATGTGACTAACTTAACC	360	(Jackson et al., 2004)
	FA2	TAATGGTGAATCTTGGTTTGG		
E. faecium ATCC 35667	FM1	GAAAAAACAATAGAAGAATTAT	215	
	FM2	TGCTTTTTTGAATTCTTCTTTA		
E. gallinarum Field strain (NHLS)	GA1	TTACTTGCTGATTTTGATTCG	173	

	GA2	TGAATTCTTCTTTGAAATCAG	
E. casseliflavus ATCC 700327	CA1	TCCTGAATTAGGTGAAAAAAC	288
	CA2	GCTAGTTTACCGTCTTTAACG	

*Field strains were provided by the National Health Laboratory Services (NHLS), South Africa.

Table S2: Enumeration of *Enterococcus* in soil and chicken litter over the sampling period

**Sample collection Round**	**Farm activities during sample collection**	**Sample collection day**	**Mean *Enterococcus* log count /Sample point (MPN/g x10⁷)**						**Geometric mean of *Enterococcus* count in soil/ Sample Round (MPN/g x10⁷)**	**Geometric mean of *Enterococcus* count in the heap of chicken litter/ Sample Round (MPN/g x10⁷)**
			A	B	C	D	E	Heap of chicken litter		
**R1**	Before litter application	Day 1	3.89	4.31	4.19	3.73	-	-	4.02	-
**R2**		Day 2 [b]	2.91	3.37	2.75	1.55	2.74	-	2.54	-
**R3**		Day 3 [r]	2.79	2.86	2.43	2.21	2.93	-	2.53	-
**R4**		Day 5	2.60	1.84	2.31	1.58	2.04	-	2.00	-
**R5**		Day 9	2.67	3.54	3.48	4.36	2.35	-	3.19	-
**R6**	Litter application	Day 0	3.19	2.44	2.90	3.18	3.98	4.13	2.95	4.05
**R7**	After litter application	Day 1	2.20	2.40	2.31	3.55	2.89	4.42	2.59	4.41
**R8**		Day 3	4.64	2.88	1.85	3.35	3.46	3.38	3.05	3.26
**R9**		Day 7 [u]	3.09	3.30	1.45	2.58	1.87	4.7	2.26	4.7

**R10**		Day 14 [r]	4.68	4.68	4.68	4.68	4.68	4.68	4.68	4.68
**R11**		Day 21	4.84	4.68	4.68	4.99	4.99	4.43	4.83	4.41
**R12**		Day 28	5.32	4.38	5.52	5.68	5.68	4.99	5.27	4.97
**R13**		Day 35	5.32	4.81	5.68	4.46	3.01	4.42	4.27	4.35
**R14**		Day 42 [r]	5.16	5.68	4.81	5.68	5.68	5.05	5.37	5.05
**R15**		Day 49	4.12	3.19	2.05	3.25	2.75	4.79	2.90	4.77
**R16**		Day 56	3.50	3.53	3.97	5.35	3.71	4.12	3.82	4.10
**R17**		Day 63	1.65	4.65	2.83	3.55	1.00 [a]	3.35	3.10	2.71
**R18**		Day 77 [r]	6.38	2.80	2.92	2.88	3.53	4.32	3.50	4.24
**R19**		Day 91	3.79	3.34	4.29	5.15	3.33	5.68	3.65	5.68
**R20**		Day 105	2.60	4.7	1.96	5.61	5.68	3.68	3.67	3.67

[a] *Enterococcus* was x 10^0; [b] The day the farm was burnt; [r] Days it rained on the farm; [u] Urea salt was applied to the soil; - Sample not collected

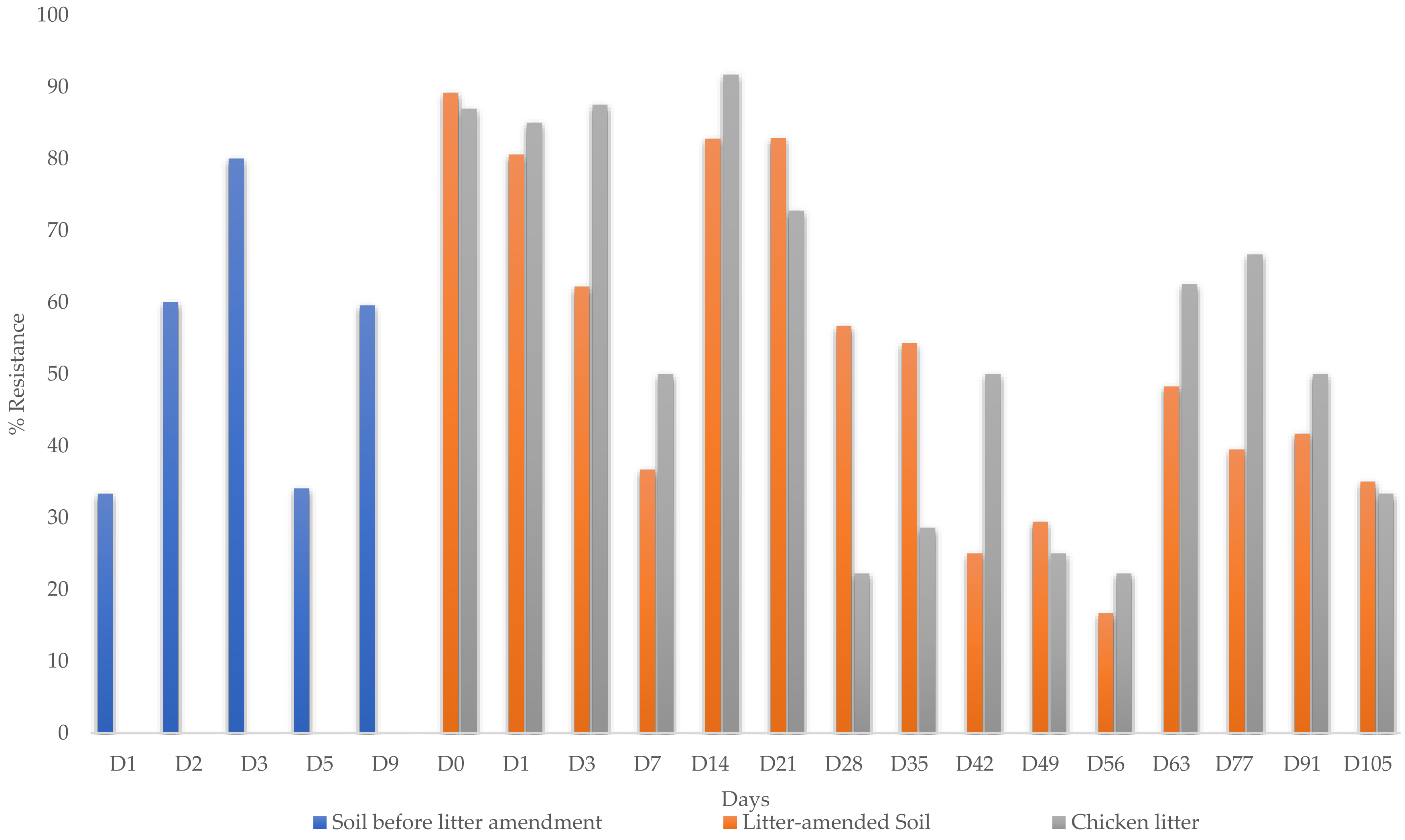

Figure S1. Variation in antibiotic-resistant *Enterococcus* to at least one antibiotic throughout sample collection.

Table S3: Multidrug-resistant pattern of the *Enterococcus* spp. isolates

**Antibiogram**	Frequency	*E. casseliflavus* (*n* = 469)	*E. faecalis* (*n* = 184)	*E. faecium* (*n* = 64)	*Enterococcus* spp. (*n* = 102)	*E. gallinarum* (*n* = 16)	**Sample source**		
							**Unamended soil (*n* = 107)**	**Litter-amended Soil (*n* = 573)**	**Chicken Litter (n = 155)**
**DAY 3 SAMPLING**									
CIP-TET-SXT	**4**	2	1	1	0	0	**1**	**1**	**2**
LEV-CIP-TET-SXT	**1**	1	0	0	0	0	**1**	**0**	**0**
**DAY 5 SAMPLING**									
ERY-TET-SXT	**31**	17	8	3	3	0	**1**	**25**	**5**
VAN-IPM-ERY-LEV-SXT	**1**	1	0	0	0	0	**1**	**0**	**0**
**DAY 7 SAMPLING**									
NIT-CIP-TET-SXT	**3**	2	0	1	0	0	**2**	**0**	**1**
VAN-LEV-TET-SXT	**1**	1	0	0	0	0	**1**	**0**	**0**
AMP-NIT-TET	**1**	1	0	0	0	0	**1**	**0**	**0**
LEV-TET-SXT	**2**	1	0	1	0	0	**1**	**1**	**0**
VAN-ERY-CIP-TET	**1**	1	0	0	0	0	**1**	**0**	**0**
VAN-CIP-TET	**1**	1	0	0	0	0	**1**	**0**	**0**
VAN-AMP-TET-SXT	**1**	1	0	0	0	0	**1**	**0**	**0**
VAN-ERY-CIP-TET-SXT	**1**	1	0	0	0	0	**1**	**0**	**0**
**DAY 0 OF MANURE APPLICATION**									
ERY-CIP-TET	**9**	5	1	1	2	0	**0**	**8**	**1**

NIT-TET-SXT	**2**	1	1	0	0	0	**0**	**1**	**1**
ERY-NIT-TET	**3**	2	1	0	0	0	**0**	**3**	**0**
TEC-VAN-ERY-NIT	**1**	1	0	0	0	0	**0**	**1**	**0**
ERY-LEV-TET	**1**	1	0	0	0	0	**0**	**1**	**0**
TEC-VAN-NIT-SXT	**1**	1	0	0	0	0	**0**	**1**	**0**
ERY-CIP-TET-SXT	**4**	2	0	1	1	0	**0**	**3**	**1**
STR-ERY-CIP-TET	**1**	0	1	0	0	0	**0**	**1**	**0**
STR-LEV-CIP-TET	**1**	0	1	0	0	0	**0**	**1**	**0**
STR-ERY-TET-SXT	**7**	0	5	1	1	0	**0**	**6**	**1**
STR-AMP-ERY-TET-SXT	**1**	1	0	0	0	0	**0**	**1**	**0**
STR-AMP-ERY-TET	**1**	0	1	0	0	0	**0**	**1**	**0**
ERY-NIT-TET-SXT	**4**	2	1	1	0	0	**0**	**3**	**1**
STR-ERY-QD-CIP-TET-SXT	**1**	0	0	1	0	0	**0**	**0**	**1**
AMP-IPM-ERY-LEV-QD	**1**	0	0	1	0	0	**0**	**0**	**1**
**DAY 1 SAMPLING AFTER MANURE APPLICATION**									
ERY-NIT-SXT	2	1	0	0	1	0	**0**	**2**	0
LEV-NIT-CIP-TET-SXT	1	1	0	0	0	0	**0**	**1**	0
AMP-TET-SXT	2	2	0	0	0	0	**0**	**1**	**1**
AMP-ERY-SXT	1	0	0	0	1	0	**0**	**1**	0
STR-AMP-IPM-ERY-TET-SXT	1	0	0	1	0	0	**0**	**1**	0
AMP-ERY-NIT-TET	1	1	0	0	0	0	**0**	**1**	0
AMP-ERY-TET	1	1	0	0	0	0	**0**	**1**	0
IPM-ERY-TET	3	1	0	1	1	0	**0**	**2**	**1**
IPM-ERY-CIP-TET	3	1	0	1	1	0	**0**	**2**	**1**

STR-IPM-ERY-CIP-TET-SXT	1	1	0	0	0	0	**0**	0	**1**
AMP-IPM-ERY-LEV-TET	1	1	0	0	0	0	**0**	0	**1**
**DAY 3 SAMPLING AFTER MANURE APPLICATION**									
VAN-ERY-TET	1	1	0	0	0	0	**0**	**1**	**0**
IPM-ERY-NIT	1	1	0	0	0	0	**0**	**1**	**0**
IPM-ERY-LEV-NIT-TET	1	0	0	1	0	0	**0**	**1**	**0**
AMP-LEV-CIP-SXT	1	0	1	0	0	0	**0**	**1**	**0**
AMP-ERY-TET-SXT	2	1	1	0	0	0	**0**	**2**	**0**
AMP-ERY-NIT-TET-SXT	1	0	0	0	1	0	**0**	**0**	**1**
ERY-QD-TET-SXT	1	0	0	1	0	0	**0**	**0**	**1**
ERY-QD-TET	2	0	0	2	0	0	**0**	**1**	**1**
**DAY 14 SAMPLING AFTER MANURE APPLICATION**									
ERY-CIP-TET-QD	1	0	0	1	0	0	**0**	**1**	**0**
IPM-CIP-TET	1	0	0	0	1	0	**0**	**0**	**1**
IPM-QD-CIP-TET	1	0	0	1	0	0	**0**	**0**	**1**
AMP-IPM-ERY-NIT	1	1				0	**0**	**0**	**1**
**DAY 21 SAMPLING AFTER MANURE APPLICATION**									
IPM-ERY-LEV-CIP-SXT	1	0	0	0	1	0	**0**	**1**	**0**
IPM-LEV-CIP-SXT	1	0	0	1	0	0	**0**	**1**	**0**
IPM-TET-SXT	1	0	0	0	1	0	**0**	**1**	**0**
NIT-QD-SXT	1	0	0	1	0	0	**0**	**1**	**0**
ERY-LEV-CIP-TET-SXT	1	1	0	0	0	0	**0**	**1**	**0**
ERY-NIT-CIP-TET-SXT	1	0	0	1	0	0	**0**	**0**	**1**

IPM-ERY-NIT-SXT	1	0	0	1	0	0	**0**	**0**	**1**
IPM-ERY-LEV-QD-CIP-TET-SXT	1	0	0	1	0	0	**0**	**1**	**0**
AMP-LEV-TET	1	1	0	0	0	0		**0**	**1**
**DAY 28 SAMPLING AFTER MANURE APPLICATION**									
STR-ERY-TET	1	1	0	0	0	0	**0**	**1**	**0**
**DAY 77 SAMPLING AFTER MANURE APPLICATION**									
STR-ERY-SXT	1	1	0	0	0	0	**0**	**1**	**0**
STR-AMP-ERY	1	1	0	0	0	0	**0**	**1**	**0**
STR-CIP-SXT	1	1	0	0	0	0	**0**	**1**	**0**
**Total 27.9% (130/466)**	**130**	**66**	**23**	**26**	**15**	**0**	**2.8% (13/466)**	**18.9% (88/466)**	**6.2% (29/466)**

microorganisms

MDPI

Table S4: Percentage of *Enterococcus* isolates that were resistant to at least one antibiotic at each sample point and the multiple antibiotic resistance index

Sampling point	Total isolates	STR	TEC	VAN	AMP	LZD	IPM	ERY	LEV	NIT	CHL	QD*	TGC	CIP	GEN	TET	SXT	MDR	MAR index
A	149	1	1	3	1	0	5	24	5	5	0	29	0	11	0	37	28	16	0.08
B	122	2	1	6	4	0	5	24	2	4	1	0	0	10	0	22	21	12	0.07
C	126	2	0	2	1	0	2	25	6	2	0	22	0	12	0	25	20	16	0.07
D	165	1	0	6	7	0	2	21	3	6	0	0	0	12	0	36	25	16	0.08
E	118	3	0	0	4	0	4	26	1	3	0	33	0	11	0	35	19	14	0.07
Heap	155	2	0	1	6	0	8	31	2	8	0	19	0	11	0	37	25	19	0.09

AMP = Ampicillin, TGC = tigecycline, TET = tetracycline, CHL = chloramphenicol, GEN = gentamicin, TEC = teicoplanin, VAN = vancomycin, STR = streptomycin, LZD = linezolid, IPM = imipenem ERY = erythromycin, CIP = ciprofloxacin, LEV = levofloxacin, NIT = nitrofurantoin, SXT = sulfamethoxazole/trimethoprim, QD = Quinupristin-dalfopristin. QD* is reported only for *E. faecium* isolates.
